# Willingness of community based health insurance uptake and associated factors among urban residents of Oromia regional state, Oromia, Ethiopia, a cross-sectional study

**DOI:** 10.1186/s12913-020-05583-x

**Published:** 2020-09-16

**Authors:** Alem Deksisa, Meyrema Abdo, Ebrahim Mohamed, Daniel Tolesa, Sileshi Garoma, Abate Zewdie, Melese Lami, Dinka Irena, Dereje Abdena, Hunde Lemi

**Affiliations:** 1 Department of Public Health, Adama Hospital Medical College, Adama, Ethiopia; 2 Oromia Regional Health Bureau, Addis Ababa, Ethiopia

**Keywords:** Willingness, Community based health insurance, Urban, Households

## Abstract

**Background:**

Globally, Millions of people cannot use health services because of the fear of payment for the service at the time of service delivery. From the agenda of transformation and the current situation of urbanization as well as to ensure universal health coverage implementing this program to the urban resident is mandatory. The aim of this study is to assess the willingness of community-based health insurance (CBHI) uptake and associated factors among urban residents of Oromia regional state, Oromia, Ethiopia, 2018.

**Methods:**

A community-based cross-sectional study was conducted. From the total of eighteen towns; six towns which account for **33%** of the total were selected randomly for the study. One population proportion formula was employed to get a total of 845 households. A pre-tested, semi-structured interviewer-administered questionnaire was used to collect the required data. Double-Bounded Dichotomous Choice Variant of the contingent valuation method was used to assess the maximum willingness to pay for the scheme, and a multiple logistic regression model was used to determine the effect of various factors on the willingness to join and willingness to pay for the households.

**Result:**

About 839 (99.3%) of the respondents participated. The mean ages of the respondents were 40.44(SD ± 11.12) years. 621 (74.1%) ever heard about CBHI with 473 (56.3%) knowing the benefits package. Out of 839, 724 (86.3%) were willing to uptake CBHI of which 704 (83.9%) were willing to pay if CBHI established in their town.

**Conclusion:**

If CBHI established about 86.3% of the households would enroll in the scheme. Having education, with a family size between 3 & 6, having difficulty in paying for health care and less than 20mins it took to reach the nearest health facility were the independent predictors of the willingness of CBHI uptake. The Oromia and Towns Health Bureau should consider the availability of health facilities near to the community and establishing CBHI in the urban towns.

## Background

Community-based health insurance schemes help to give financial protection and decrease direct out- of-pocket payment for health care based on the assumption of risk-pooling and community solidarity to risks of falling sick [1]. CBHI schemes allow people’s resources to be pooled to cover the costs of unpredictable health problems and keep individuals and households from the risk of catastrophic medical expenses in exchange for out-of-pocket payments [2, 3].

To achieve universal coverage for health care; government and donor agencies in a number of developing countries are not implementing Community-based health insurance schemes (CBHI) as social protection and an alternative measure. Community-based health insurance schemes are becoming increasingly recognized as one of health care financing strategy in developing country [4].

The health sector transformation plan (HSTP) of Ethiopia has put very motivating goals and desires to renovate the health system to deliver equitably and quality health cares. It is the first the envisage of stage of Ethiopia path towards universal health coverage through strengthening primary health care and as part of the second growth and transformation plan (GTP II) of the country. The main programs of HSTP are ensuring equity and quality health care services, the information revolution, woreda (the third-level-administrative divisions of Ethiopia) transformations, and caring, respectful and compassionate health workforce [5].

The three goals of woreda transformation program are: developing high-performing primary health care units (PHCU), the graduation of model Kebeles (the smallest unit of local government in Ethiopia), and achievement of universal health coverage with financial risk protection; which focuses on CBHI [6].

After successful completion of the 20-years health sector development program (HSDP); the Government of Ethiopia developed a road map in which the health sector envisioned beyond strengthening primary health care unit was placed as a strategy. Health sector transformation plan is part of this strategy which is implemented from the Ethiopian fiscal year 2008 to 2012 (July 2015 – June 2020) [5, 7].

As part of health care financing strategy, the government of Ethiopia endorsed and launched CBHI scheme in 13 pilot woreda in Amahara, Oromia, south nation nationalities peoples and Tigray regions in 2010/11 to provide a risk protection mechanism for those employed in the rural and the informal sectors. The 13 pilot woredas are still implementing CBHI and they are on the way to expand it to 185 woredas [8].

Community-Based Health Insurance (CBHI) scheme in Oromia began in 2001 Ethiopian fiscal year (EFY) in four zones and four woredas and currently expanded into 134 additional woredas distributed among all zones. The objective of the scheme is to improve health service utilization by reducing direct out of pocket payments and improve the quality of health services [9].

Even though enormous activities were undertaken to guide and support the program since the regional cabinet passed a decision to expand the program, still the performance was very low ranging from 23% enrolment rate in the pilot woredas to 25% in the two phase expansions of 71 woredas [10].

In the current study area, there are no published data on demand of CBHI. It is believed that this study will help policy makers to address factors which affect the HHs willingness to uptake (WTU) make the benefit of planned CBHI scheme. The objective of this study was to assess willingness of community based health insurance uptake (WCBHIU) and associated factors among urban towns of Oromia region, Ethiopia, 2018.

## Methods

### Study design and setting

The community-based cross-sectional study design was conducted. The study was conducted in six towns of Oromia regional state from May 26 to July 30, 2018. Oromia regional state is the first populous and broader state among the nine regional states of Ethiopia. It’s bordered with all regional states except Tigray regional state and has two international borders Kenya with South and South Sudan with West. Administratively, Oromia regional state is composed of twenty zones and eighteen towns which are subdivided into 333 woredas (the smaller administrative unit) and 7011 Kebeles (the smallest administrative unit). Projections from the 2007 population and housing census estimate the total population for the year 2017/18 to be 36,839,051 with the sex ratio between males and female is almost equal(1:1) and average annual population growth rate of 2.9% (%2.7 & %4.6 in rural and urban respectively). On average 123 populations live per Sq. Km and there is a variation from one zone to another zone. In the year 2017, there are 79 hospitals, 1366 health centers, 6559 health posts, 2 regional laboratory and 7 blood bank unit government health facilities and also 7 private hospital, 150 private health centers, different level 3149 private clinics and 1701 pharmacies, 8 and 5 government development organization hospitals, and health centers respectively providing health services in the region. The health service coverage of the region is 97% & 98% by health centers and health post respectively.

### Sample size determination

The sample size was calculated using a single population proportion formula by “Taro Yamane” [11], as follows;

$$ n=\left(Z\propto /2p\left(1-p\right)\right)/{d}^2 $$

P = expected rate of willingness to join a community-based health insurance scheme = 0.5 (since there is no Urban willingness to join study conducted in the country so far)

$$ {\displaystyle \begin{array}{l}\mathbf{d}=\mathrm{Margin}\ \mathrm{of}\ \mathrm{sampling}\ \mathrm{error}\ \mathrm{tolerated}=5\%\\ {}=\mathrm{Critical}\ \mathrm{value}\ \mathrm{at}\ 95\%\mathrm{confidence}\ \mathrm{interval}\ \mathrm{of}\ \mathrm{certainty}\ (1.96)\\ {}=(1.96)2\ast 0.5\ \left(1--0.5\right)/(0.05)2=384\end{array}} $$

By adding the expected non-response rate of 10%, the sample size was 290HHs, but since the study utilized multi-stage sampling, this sample size was multiplied by 2 for the design effect. Hence, the final sample sizes for this study is 2 *384 + 10% = 845.

### Study population

**Fig. 1:**
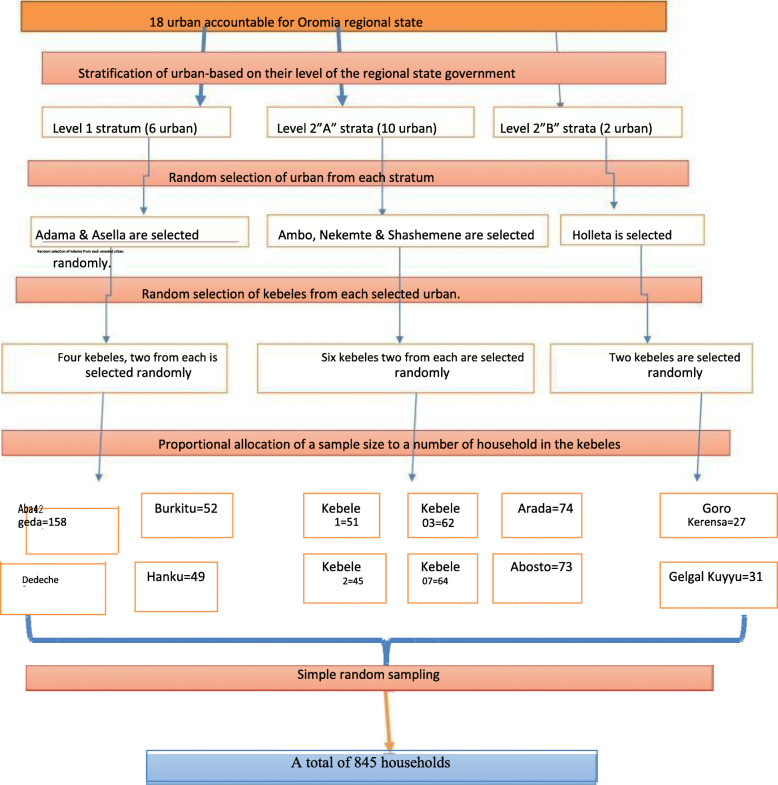
Schematic presentation of sampling procedure, Oromia regional state, 2018, Adama, Ethiopia

The sample was obtained using stratified multi-stage simple random sampling technique. Eighteen selected towns were stratified into three strata based on their rank given by the regional state government. Two towns were selected randomly from the first level and from 2″ A” level three and one from 2 “B” level. Finally, six towns from all levels were selected randomly from all towns in the region. Twelve kebeles (the smallest administrative division); two from each were selected using lottery methods from all selected study towns. In the second stage, 845 households were selected using computerized simple random sampling technique. The households who are selected to participate in the study were allocated proportionately to the size of households of those kebeles. The sampling frame was developed by using the identification number of the houses which were given by the kebeles (Fig. 1).

### Data collection

Data were collected using an Interviewer administered pretested, a structured and standardized questionnaire by 12 trained data collectors. The questionnaire was adapted from previous similar studies for data collection purpose [3, 7, 9] and. It was initially prepared in English and then was translated into Afan Oromo (the local language) and later on back to English to check for consistency. Supervisors followed the data collectors and provided any necessary correction on the spot.

‘Double-bounded dichotomous choice variant of the contingent valuation method’ was used in which respondents were asked two successive binary questions for their ability to pay the stated number of premiums for CBHI. First the respondents were asked their ability to pay 500ETB initial bid per year per household for CBHI. The second question was conditioned on the responses to the first answer. That means if the response was “yes, they were asked their ability to pay double of the first bid (premium) for CBHI. If their responses to initial bid was “no,” they were asked for their ability to pay half of the initial bid. Finally, open-ended question for those who did not pick a ‘yes’ for either the first or second option was used to enable respondents to pick lower amounts (as low as zero) or higher amounts (higher than the stated options in the double bounded dichotomous choice contingent valuation Method).This helped the researchers to separate those respondents whose real ability to pay were zero from those respondents who were willing to pay something but less than the lowest bid.

### Data analysis

The collected data were cleaned, coded and entered into EPI Data version 3.1 and then exported for analysis to SPSS version 20. The data was analyzed using binary and multivariate logistic regressions to determine the effect of various factors on the outcome variable. The results were presented in the form of tables, figures and text using frequencies and summary statistics such as standard deviation, mean, and percentage to describe the study population in relation to relevant variables. The degree of association between dependent and independent variables was assessed using odds ratio with 95% confidence interval and *p*-value < 0.05 declared statistical significance.

## Results

### Socio-demographic characteristics of respondents

A total of 845 participants from six urban towns of Oromia was planned to participate in the study, out of which 830 study subjects were enrolled; with 322 (39.2%) from Adama, 101 (12.1%) from Asella, 94 (11.3%) from Ambo, 108 (13.2%) were from Nekemte, 147 (18%) were from Shashemene and the rest 6.2% from Holleta town; making a response rate of 98.2%. The reason for non-participation was unwillingness.

Among study participants 315 (38%) were in the age group of 35–44 years with median 38 years. (interquartile range of 32 to 46 years). 449 (54.4%) were male. 438 (52.8%) were husbands by a head to the household followed by 366 (44.1%) spouses by a head to the household. About 424 (51.1%) were orthodox by religion, and 659 (79.4%) were married. By occupation 288 (34.7%) were merchants and 293 (35.2%) were educated within grade 9–12. The average family size of the household was 3–6 for 455 (54.8%) of the participants (Table 1).

**Table 1:** Distribution of socio-demographic characteristics of respondents who participated in the survey, urban towns of Oromia, 2018, Ethiopia

Variables	Frequency	Percent (%)
Age category		
15–24	25	3.0
25–34	221	26.6
35–44	315	38.0
45–54	168	20.2
> 54	101	12.2
Sex		
Male	449	54.1
Female	381	45.9
Relation to the head of the HH		
Husband	438	52.8
Spouse	366	44.1
Child	18	2.2
Others	8	1.0
Religion		
Orthodox	424	51.1
Muslim	141	17.0
Protestant	246	29.6
Catholic	18	2.2
Others	1	.1
Ethnicity		
Amhara	188	22.7
Oromo	488	58.8
Guragie	97	11.7
Tigre	37	4.5
Others	20	2.4
Marital status		
Single	49	5.9
Married	659	79.4
Divorced	46	5.5
Widowed	74	8.9
Separated	2	.2
Occupation		
Farmer	43	5.2
Housewife	213	25.7
Merchant	288	34.7
Daily Laborer	167	20.1
Priv. Com. Employee	3	.4
Self-employed	15	1.8
Others	101	12.2
Educational status		
Can’t read and write	43	5.2
Read and write	69	8.3
Grade 1–8	204	24.6
Grade 9–12	293	35.3
Certificate/Diploma	178	21.4
Degree and above	43	5.2
Family size		
< 3	296	35.7
3–6	455	54.8
> 6	79	9.5
Pregnancy		
Yes	154	18.6
No	676	81.4
Participation in Iddir		
Yes	630	75.9
No	200	24.1

### Social capital and horizontal trust

Almost half of the respondents 422 (50.8%), 426 (51.3%), 420 (50.6%), 408 (49.2%), 409 (49.3%), 430 (51.8%) and 408 (49.2%) disagreed with the statements; ‘most villagers of the village can be trusted’, ‘Most villagers willing to return; what doesn’t belong to them’, ‘Neighbors can be trusted’, ‘Village leaders are trusted’, ‘Villagers concern issues not only relate to themselves’, ‘Villagers provide help if someone really needs it and ‘Lend money to your neighbors’, respectively. About 381 (45.9%), 332 (40%) and 489 (58.9%) disagreed with the statements: ‘Most villagers of the village try to take advantage’, ‘Were Village had a large Family would be a member of this family and I would like to support a project that might benefit other villagers, respectively. A total of 715 respondents disagreed with overall horizontal trust statements (Table 2).

**Table 2:** Distribution of social capital and horizontal trust among respondents, Oromia, 2018, Ethiopia

Variables			Frequency	Percent
**Most villagers of the village can be Trusted**	Strongly agree	14		1.7
	Agree	87		10.5
	Neutral	19		2.3
	Disagree	422		50.8
	Strongly disagree	288		34.7
**Most villagers of the village try to take Advantage**	Strongly agree		20	2.4
	Agree	95		11.4
	Neutral	37		4.5
	Disagree	381		45.9
	Strongly disagree	297		35.8
**Most villagers willing to return; what doesn’t belong to them**	Strongly agree	12		1.4
	**Agree**	**94**		11.3
	Neutral	26		3.1
	**Disagree**	**426**		51.3
	Strongly disagree	272		32.8
**Neighbors can be Trusted**	Strongly agree	2		.2
	Agree	50		6.0
	Neutral	19		2.3
	Disagree	420		50.6
	Strongly disagree	339		40.8
**Village leaders are Trusted**	Strongly agree	90		10.8
	Agree	142		17.1
	Neutral	73		8.8
	Disagree	408		49.2
	Strongly disagree	117		14.1
Villagers concern issues not only relate to themselves	Strongly agree	28		3.4
	Agree	98		11.8
	Neutral	25		3.0
	Disagree	409		49.3
	Strongly disagree	270		32.5
Villagers provide help if someone really needs it	Strongly agree	7		.8
	Agree	58		7.0
	Neutral	29		3.5
	Disagree	430		51.8
	Strongly disagree	306		36.9
**Lend money to your Neighbors**	Strongly agree	3		.4
	Agree	45		5.4
	Neutral	33		4.0
	Disagree	408		49.2
	Strongly disagree	341		41.1
**Were Village had a large** **A family would be a member of this family**	Strongly agree	111		13.4
	Agree	108		13.0
	Neutral	31		3.7
	Disagree	332		40.0
	Strongly disagree	248		29.9
**I Would like to support a project that might benefit other villagers**	Strongly agree	29		3.5
	Agree**a**	80		9.6
	Neutral	30		3.6
	Disagree	489		58.9
	Strongly disagree	202		24.3
**Overall horizontal**	Agree	115		13.9
**Trust**	Disagree	715		86.1

### Health and health-related factors

Majority of the respondents 380 (45.8%) said the health status of their family is good. Only 95 (11.4%) had a chronic illness and/or disability. 485 (58.4%) of respondents and 516 (62.2%) of respondents family members had encountered an illness in the last 12 months. Latest illness Episode 142 (17.1%) occur within the last 1 month followed by 136 (16.4%) occurring within 7–12 months. Of which 501 (60.4%) got treatment from different facilities and 216 (26%) from private health facility; as 180 (21.7%) chose for its service is efficacious. The total health care cost for the last 12 months was less than 1000 ETB for 386 (77%) of the respondents; 481 (96%) said it’s covered by themselves and getting that money was very difficult for 197 (39.2%) of them. And 353 (42.5%) responded that it took 10–20 min to reach the health facility. To 326 (39.3%) of the respondents, a private clinic is the nearest followed by health center for 206 (24.8%). 437 (52.7%) can raise 200 Ethiopian birr (ETB) within a week in case of emergency and it’s from their own cash for 242 (55.4%) of them. A merchant is the main source of income for 323 (39%) of the respondents (Table 3).

**Table 3:** Distribution of health and health-related factors among respondents, Oromia, 2018, Ethiopia

Health status of the	Very poor	17	2.0
**Family**	Poor	31	3.7
	Medium	251	30.2
	Good	380	45.8
	Very good	151	18.2
**Have chronic illness and/or disability**	Yes	95	11.4
	No	735	88.6
**You encountered any illness in the last 12 months**	Yes	485	58.4
	No	345	41.6
**member of the family encountered any illness during the last 12 months**	Yes	516	62.2
	No	314	37.8
Latest illness episode Occur	Before one year	50	6.0
	Within the last 7–12 Months	136	16.4
	Within the last 4–6 Months	117	14.1
	Within the last 2–3 Months	71	8.6
	Within the last 1 Month	142	17.1
	Total	516	62.2
**Get treatment Facility**	Yes	501	60.4
	No	15	2.0
	Total	516	62.4
	home treatment	11	1.3
	Local drug vendor	6	.7
	Private Health Facility	216	26.0
	Public health center	110	13.3
	Public hospital	150	18.1
	Traditional healer	8	.9
	Total	501	60.4
**Reason to go to HF**	The HF was physically accessible	111	13.4
	The HF was not expensive	158	19.0
	The health facility not too crowded	21	2.5
	The health service was courteous	31	3.7
	The health service was efficacious/Effective	180	21.7
	System	329	39.6
**Reason for not getting Rx**	Considering the illness is self-Limiting	10	1.2
	No enough money	7	.8
	Total	17	2.0
	System	813	98.0
**No of illness**	< 2	477	92.4
	2–4	36	7.0
	> 4	3	.6
	Total	516	100.0
**Total HC cost**	< 1000	386	77.0
	1000–3000	87	17.4
	> 3000	28	5.6
	Total	501	100.0
**Time to reach HF in Min**	< 10 min	262	31.6
	10-20 min	353	42.5
	> 20 min	215	25.9
**Variables**		Frequency	Valid Percent
**HC cost covered by**	Self	481	96.0
	Government/free	3	.6
	Community	5	1.0
	Others	12	2.4
	Strongly agree	98	19.5
**Your satisfaction with health care service in wliinthe the costs**	Agree	79	15.7
	Neutral	77	15.3
	Disagree	157	31.2
	Strongly disagree	90	18.3
	Total	501	100.0
**Getting money to pay fthore health Care**	Very difficult	197	39.2
	Difficult	161	32.1
	Not difficult	144	28.7
	Total	502	100.0
**Covered HC by**	Self	481	96.0
	Government/free	3	.6
	Community	5	1.0
	Others	12	2.4
	Total	501	100.0
**Borrow money from**	Yes	153	18.4
**Relatives**	No	677	81.6
**Nearest HF to home**	Health center	206	24.8
	Clinic (Private)	326	39.3
	private hospital	90	10.8
	Hospital (Gov.)	176	21.2
	Non-Gov’tal health facilities	32	3.9
**Raise 200 Birr within a week In case of emergency**	Yes	437	52.7
	No	393	47.3
**HH obtain the 200**	sale of the animal.	14	3.2
**ETB from**	and animal product		
	sale of crops	27	6.2
	sale of forest products	6	1.4
	own cash	242	55.4
	bank saving	95	21.7
	Equb	4	.9
	Iddir	5	1.1
	loan from a bank or other institutions	6	1.4
	loan from relatives	6	1.4
	gifts from relatives	2	.5
	loan from non- relatives	7	1.6
	sale of household assets	11	2.5
	sale of personal item (Jewelries, etc.)	7	1.6
	Other (specified)	5	1.1
	Total	437	100.0
**The main source of Income**	Merchant	323	39
	Pension	43	5.2
	Selling Injera	125	15
	Grocery	62	7.5
	Renting house	47	5.7
	Private work	113	13.6
	Sewing clothes	59	7
	Daily laborer	33	4
	Others	25	3

### Awareness, willingness to uptake and ability to pay for CBHI

**Fig. 2:**
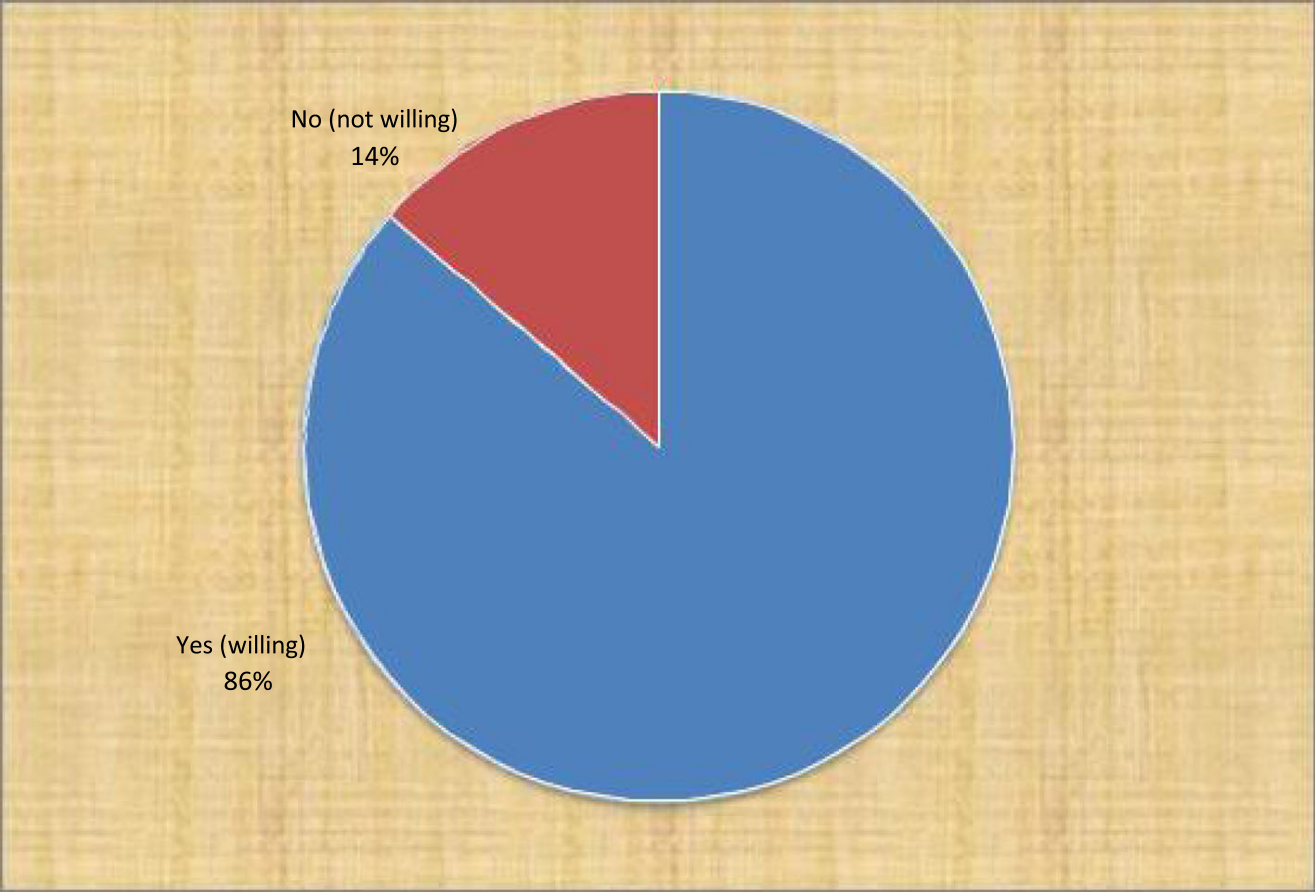
Willingness of CBHI uptake if established in urban towns of Oromia, 2018, Adama, Ethiopia

From total respondents 619 (74.6%) had heard about CBHI; from these 473 (57%) know the benefits package of CBHI. Among respondents 716 (86.3%) were willing to uptake CBHI; out of this 688 (83.9%) were willing to pay, and 415 (58%) were able to pay 500ETB as annual premium for CBHI. The reason to uptake CBHI for 259 (36.2%) of the participants was for security and peace of mind in times of ill-health. About 50 (46.3%) out of 108 respondents not willing to uptake CBHI, were as a result of not having enough money and around 30 (46.9%) out of 64 respondents not willing to pay for CBHI, was due to a shortage of money. The study indicated that 278 (39.3%) wanted to pay the premium bi-annually followed by 230 (32.5%) wanting to pay annually (Table 4), (Fig. 2).

**Table 4:** Distribution of awareness on CBHI, willingness to join CBHI and ability to pay for CBHI in Oromia Region, 2018, Ethiopia

Variables		Frequency	Percentage
**Ever heard about CBHI**	**Yes**	**619**	**74.6**
	No	211	25.4
**Know the benefits package of CBHI**	Yes	473	57
	No	237	28.6
**Benefits package of CBHI**	Drugs	320	38.6
	Surgery except for cosmetic Surgery	67	8.1
	Inpatient stay	89	10.7
	Laboratory Tests	43	5.2
	Others	8	1.0
**Heard CBHI from**	Radio	219	26.4
	HEW	29	3.5
	TV	289	34.8
	Neighbor	37	4.5
	Leader of HAD	42	5.1
	Others	2	.2
	Missing	212	25.5
**Variables**		**Frequency**	**Percent**
**Willing to**	**Yes**	716	86.3
**uptake CBHI**	**No**	114	13.7
**Reason to uptake CBHI**	It provides free access to medical care	243	33.9
	To help others	68	9.5
	For security and peace of mind in times of ill- Health	259	36.2
	Facing health problem	15	2.1
	Frequently unable to cover medical care cost at the time of ill-health	117	16.3
	Other	14	2.0
Reason for not join the scheme	not have enough money to pay	50	46.3
	Do not need health insurance	34	31.5
	Other	24	22.2
**Willing to pay for CBHI**	Yes	688	96.2
	No	28	3.8
Can pay 500ETB/year	Yes	415	58.0
	No	301	42.0
Pay the initial bid as annual premium/HH for CBHI	Yes	404	64.2
	No	225	35.8
Pay if the premium is double	Yes	165	27.9
	No	426	72.1
Pay if the premium is halved	Yes	362	74.2
	No	126	25.8
Max. pay/year as a premium	< 500	397	47.3
	500–1000	114	13.6
	> 1000	66	7.9
The reason the HH not willing to pay for the scheme	Doubt the management of the fund	10	15.6
	Because of lack of money	30	46.9
	Out-of-pocket payment is better than CBHI scheme	17	26.6
	Others	7	10.9
Frequency want to pay the yearly premium	Annual flat rate	230	32.5
	Bi-annual flat-rate	278	39.3
	Quarterly a year flat-rate	144	20.3
	Monthly	46	6.5
	Others	10	1.4

### Risk factors for the willingness of CBHI uptake

Multivariable logistic regression analyses were conducted to explore the association between dependent and independent variables. So, the study showed that the odds of willingness to utilize CBHI are associated with educational status, family size, easiness of getting money to pay for health care services; the time it took to reach the nearest health facility and frequency at which the respondents want to pay the yearly premium.

Accordingly having a certificate/diploma, learning from grade 1–8 and able to read and write were 3.38 (AOR 3.38; 95% CI: 1.27, 8.98), 2.90 (AOR 2.9; 95% CI: 1.16, 7.30) and 3.84 (AOR 3.84; 95% CI: 1.23, 12.01) times higher odds compared to can’t read and write, respectively; after controlling for other effects. Respondents had a family size of 3–6 had higher odds of willingness to utilize CBHI compared to with the family size of less than three (AOR = 1.95, 95% CI: 1.21–3.15). The odds of the willingness of CBHI uptake among respondents with very difficult and difficult in getting money to pay for the health care were 82 and 83% less than those without difficulty (AOR = 0.18, 95% CI: 0.07–0.49) and (AOR = 0.17, 95%CI: 0.06–0.46), respectively. Time to reach the nearest health facility in < 10 min and 10–20 min were 63 and 75% less likely willing to join CBHI when compared to the time it took > 20 mins. Ability to raise 200ETB during emergency had an association with willingness to uptake CBHI (COR = 2.02, 95% CI: 1.35–3.03); but it doesn’t have an association when adjusted (Table 5).

**Table 5:** Risk factors for the willingness of CBHI uptake in survey data in urban towns in Oromia region, 2018, Ethiopia

Variables	Willingness to join CBHI		Crude OR (95% CI)	Adjusted OR (95% CI)
	Yes (%)	No (%)		
Age of the respondents				
15–24	19	6	1	1
25–34	180	41	1.39 (0.52,3.69)	0.40 (0.70, 2.28)
35–44	274	41	2.11 (0.70,5.59)	0.52 (0.09, 2.99)
45–54	157	11	4.51 (1.50,13.58)**	1.04 (0.17, 6.49)
> 54	86	15	1.81 (0.62,5.27)	0.38 (0.06, 2.39)
Educational status				
Can’t read and Write	33	10	1	1
Can read and write	63	6	3.18 (1.06, 9.52)	3.84 (1.23, 12.01)*
Grade 1–8	184	20	2.79 (1.20, 6.49)	2.90 (1.16, 7.30)*
Grade 9–12	236	54	1.26 (0.58, 2.70)	1.38 (0.58, 3.33)
Certificate/diploma	161	17	2.87 (1.21, 6.82)	3.38 (1.27, 8.98)**
1st degree and above	39	4	2.96 (0.85, 10.30)	2.79 (0.17, 10.87)
Family size				
< 3	238	58	1	1
3–6	410	45	2,22 (1.46, 3.38)	1.95 (1.21, 3.15)**
> 6	68	11	1.51 (0.75, 3.03)	1.21 (0.55, 2.68)
Getting money to pay for health care				
Very difficult	164	33	0.23 (0.10, 0.55)	0.18 (0.07, 0.49)***
Difficult	132	29	0.25 (0.12, 0.59)	0.17 (0.06, 0.46)***
Not difficult	137	7	1	1
Time to reach nearest health facility				
< 10mins	217	45	0.69 (0.40, 1.18)	0.37 (0.17, 0.80)**
10-20 min	305	48	0.52 (0.30, 0.91)	0.25 (0.11, 0.58)***
> 20mins	194	21	1	1
Able to raise 200ETB during emergency				
Yes	394	43	2.02 (1.35, 3.03)**	1.06 (0.58, 1.97)
No	322	71	1	1
Know the benefits package of CBHI				
Yes	426	47	2.49 (1.61, 3.83)**	2.78 (0.61, 10.77)
No	186	51	1	1
can pay 500ETB as annual premium				
Yes	400	4	21.62 (7.62, 61.32)*	1.08 (0.47, 17.56)
No	185	40	1	1

NB: * = significant, *p < 0.05, **p < 0.01 and ***p < 0.001

## Discussion

Community-based health insurance (CBHI) is one of the ways to provide health insurance for the informal sector and the rural populace. CBHI, in spite of its problems relating to the extent of resource pooling, has been shown to facilitate and improve access to healthcare services especially among children, pregnant women and the elderly.

The overall aim of this study was to assess the willingness of CBHI uptake and associated factors among the informal sector workers in urban towns. The proportion of willingness of CBHI uptake was 86.3%. Educational status, family size, easiness of getting money to pay for health care services; the time it took to reach the nearest health facility and frequency at which the respondents want to pay the yearly premium were the associated factors for the willingness of CBHI uptake.

Age of the respondents, wealth, knowing the benefits package of CBHI and ability to pay 500 ETB (USD $17.9) as annual premium were variables having a statistically significant association with the willingness of CBHI uptake in crude; but have no association when adjusted. Age with 45–54 categories was 4.51 (COR = 4.51: 95%CI, 1.5–13.58) times more likely willing to uptake CBHI than 15–24 age category. Those able to raise 200 ETB (USD $7.14) during the emergency were 2.02 times more likely to enroll in CBHI than their counterparts (COR = 2.02: 95%CI, 1.35–3.03). Knowing the benefits package of CBHI make the respondents wish to join CBHI (COR = 2.49: 95%CI, 1.61–3.83). And an ability to pay 500 ETB (USD $17.9) as annual premium made them will to uptake CBHI (COR = 21.62: 95%CI, 7.62–61.32).

The willingness of CBHI uptake of this survey is similar to a previously conducted survey in Cameroon 86.2% [12]. This proportion finding is higher than research conducted in Ecuador 69.3% [13]. This high discrepancy may be related to methodological issues and differences in the study areas. But, the current finding is less than that found in 2004 in Ethiopia, in which the probability of willingness to join the scheme was 94.7% [14]. The reason may be attributed to differences in the study areas and time of the study.

Having an educational level of a certificate/diploma were 3.38 times more likely to uptake CBHI, (AOR = 3.38: 95% CI, 1.27–8.98). This finding is supported by another study, in which persons having higher education level were willing to uptake CBHI [15]. It is also supported by a study conducted in Osun State, Nigeria where people with low level of education were less willing to join CBHI [16].

In this finding, respondents with 3–6 family sizes were about two times more likely willing to join CBHI than with less than three family sizes. This finding was also supported by other findings, in which respondents having a large family had a positive association with willingness to uptake CBHI [17, 18].

Different researches showed that, the wealth or socioeconomic standing of households and individuals is associated with the uptake of CBHI [11, 17]. A similar finding was also observed in India that; wealth was associated with uptake of CBHI [18]. Our study supported the above finding in which a respondent with difficulty in paying for the health care was 82% less likely to uptake CBHI compared to those with no difficulty. From this premise, it is conceivable to find that the poor are unwilling to uptake the scheme.

In terms of time taken to reach the nearest health facility within 20 min was also found to affect enrolment to CBHI. This finding was supported by other studies conducted in low and middle-income countries [16].

### Limitation of the study

The Contingent Valuation Method has the limitation of testing consumers’ demand; that means CVM cannot approve whether the consumer actually pays the number of premiums that they said for the study and the study only shows the temporal link between dependent and independent variables.

Double-bounded dichotomous choice contingent valuation method may result in inflated value because respondents may say “yes” for the amount of money they will be asked to pay and it has starting point bias.

As the study employed an interviewer administered questionnaire that might result social desirability and recall bias.

## Conclusions

Despite the above limitations, in urban towns of Oromia regional state, if CBHI established about 86.3% of the households would enroll in the scheme. Having education, with a family size between three & six, and less than 20 min it took to reach the nearest health facility were positively associated with the odds of willingness of CBHI uptake, but having difficulty in paying for health care was negatively associated with the odds of willingness of CBHIU.

## Data Availability

Data and materials are available and can be shared by the corresponding author.

## Abbreviations

CBHF: Community-Based Health Financing
CBHI: Community-Based Health Insurance
CBHIU: Community-Based Health Insurance Uptake
CHI: Community health insurance
EHSFR: Ethiopia health sector financing reform.
ETB: Ethiopian Birr
FMOH: Federal ministry of health
HH: Household
HSFRP: Health sector financing reform program
LMIC: Low and middle-income country
MINS: Minutes
OOP: Out-of-pocket payment
ORHB: Oromia regional health bureau
PCA: Principal component analysis
SPSS: Statistical software for social science
USD: United States dollar
WHO: World Health Organization
WTJ: Willingness to join
WTP: Willingness to pay
WTU: Willingness to Uptake
